# Malnutrition and Disability: A Retrospective Study on 2258 Adult Patients Undergoing Elective Spine Surgery

**DOI:** 10.3390/medicina61030413

**Published:** 2025-02-26

**Authors:** Matteo Briguglio, Andrea Campagner, Francesco Langella, Riccardo Cecchinato, Marco Damilano, Pablo Bellosta-López, Tiziano Crespi, Elena De Vecchi, Marialetizia Latella, Giuseppe Barone, Laura Scaramuzzo, Roberto Bassani, Andrea Luca, Marco Brayda-Bruno, Thomas W. Wainwright, Robert G. Middleton, Giovanni Lombardi, Federico Cabitza, Giuseppe Banfi, Pedro Berjano

**Affiliations:** 1 IRCCS Orthopedic Institute Galeazzi, Laboratory of Nutritional Sciences, 20157 Milan, Italy; 2 IRCCS Orthopedic Institute Galeazzi, Scientific Direction, 20157 Milan, Italy; 3 IRCCS Orthopedic Institute Galeazzi, GSpine 4, 20157 Milan, Italy; 4 Department of Physiotherapy, Universidad San Jorge, 50830 Villanueva de Gállego, Spain; 5 IRCCS Orthopedic Institute Galeazzi, Intensive Care Unit, 20157 Milan, Italy; 6 IRCCS Orthopedic Institute Galeazzi, Laboratory of Clinical Chemistry and Microbiology, 20157 Milan, Italy; 7 IRCCS Orthopedic Institute Galeazzi, Spine Unit 1, 20157 Milan, Italy; 8 Fondazione Policlinico Universitario Agostino Gemelli—IRCCS, UOC Chirurgia Vertebrale, 00136 Rome, Italy; 9 IRCCS Orthopedic Institute Galeazzi, Spine Unit 2, 20157 Milan, Italy; 10 IRCCS Orthopedic Institute Galeazzi, Spine Unit 3, 20157 Milan, Italy; 11 Orthopaedic Research Institute, Bournemouth University, Bournemouth BH12 5BB, UK; 12 University Hospitals Dorset, NHS Foundation Trust, Bournemouth BH7 7DW, UK; 13 IRCCS Orthopedic Institute Galeazzi, Laboratory of Experimental Biochemistry and Molecular Biology, 20157 Milan, Italy; 14 Department of Athletics, Poznań University of Physical Education, Strength and Conditioning, 61-871 Poznań, Poland; 15 Department of Computer Science, University of Milano-Bicocca, Systems and Communication, 20126 Milan, Italy; 16 Faculty of Medicine and Surgery, Vita-Salute San Raffaele University, 20132 Milan, Italy

**Keywords:** nutritional disorders, nutritional deficiency, undernutrition, dietary supplement, patient outcome assessment, prehabilitation, orthopaedic procedure, spinal fusion, arthrodesis

## Abstract

*Background and Objectives*: Malnutrition’s prevalence and its relationship with functional ability in patients with end-stage spine pathologies, i.e., any disease of the vertebral bodies, intervertebral discs, and associated joints requiring surgical intervention, are yet to be explored. This retrospective study aimed to investigate the association between malnutrition, disability, and physical health in patients undergoing elective spine surgery in our Italian hospital. *Materials and Methods*: Data between 2016 and 2019, recorded at pre-admission visits, were extracted from our institutional spine registry (ClinicalTrials.gov number: NCT03644407), excluding minor patients or those undergoing emergency or oncological surgery. The measures were the Oswestry disability index (ODI) and the physical health (PH) summary of the 36-item Short-Form Health Survey. Clinical data were linked to nine laboratory parameters from pre-operative routine blood tests, and equations to ascertain the risk of malnutrition and its diagnosis were attributed. *Results*: The study sample included 2258 spine patients (58.15% females) who underwent surgery in our Italian hospital. The ODI and PH significantly varied across body weight difference (BWd) strata in younger adults (adjusted-*p* = 0.046, η^2^ = 0.04; adjusted-*p* = 0.036, η^2^ = 0.06) and adults (adjusted-*p* = 0.001, η^2^ = 0.02; adjusted-*p* = 0.004, η^2^ = 0.02). Protein malnutrition with acute/chronic inflammation (PMAC) in both adults (adjusted-*p* < 0.001, η^2^ = 0.04; adjusted-*p* < 0.001, η^2^ = 0.04) and older adults (adjusted-*p* = 0.010, η^2^ = 0.04; adjusted-*p* = 0.009, η^2^ = 0.05) had also a discernible impact in determining the ODI and PH. In older adults, the ODI was associated with iron deficit malnutrition (IDM) (adjusted-*p* = 0.005, η^2^ = 0.06) and both the ODI and PH were associated with vitamin B deficit (VBD) (adjusted-*p* = 0.037, η^2^ = 0.01; adjusted-*p* = 0.049, η^2^ = 0.01). Trend monotonicity was diagnosis- and sex-specific, with meaningful ordered patterns being observed mostly in young males and older females. *Conclusions*: Functional ability showed an association with malnutrition in younger adults and adults when using BWd, in adults and older adults when using PMAC, and in older adults when using IDM and VBD. The authors advocate for the inclusion of nutritional management in the pre-operative evaluation to potentially enhance recovery after spine surgery.

## 1. Introduction

Spine surgery is used to treat disorders of the spine, including end-stage spine arthritis, damaged discs, slipping vertebrae, the narrowing of the space within the spinal canal, curvatures and rotations of the column, or unstable fractures. Axial and/or radicular pain and the consequences of the spine condition negatively impact patients’ daily activities [1]. Routinely, not routinely, or suggested evaluations at pre-admission pave the way to surgery depending on specific subject characteristics. Most recent assessments include health questionnaires, which bring the perspective of the patient (patient-reported outcome measures, PROMs) into the diagnostic–therapeutic journey. These tools allow clinicians and researchers to appreciate the impact of the illness on functional ability and, therefore, to examine the recovery process in a context that is important for the patient [2]. The Oswestry disability index (ODI) is one of the most prominent measures that is unique to both conservative and surgical spine care; it quantifies personal care, movements, sleeping habits, and social life [3]. Condition-specific tools like the ODI are often administered together with other questionnaires that provide information on the general health status, with the best known being the 36-item Short-Form Health Survey (SF-36) [4]. The functional capacity needed for daily living activities can also be undermined by a state of malnutritional [5].

Malnutrition is a clinically relevant, multifaceted condition whose etiology lies either in the failure of the individual to meet their nutritional requirements, hypo- or hyperphagia, or in the unspecified alteration of the nutritional status due to ailments or medications. Malnutrition is known to negatively affect the resilience for proper physical recovery after surgery [6]. Considering the phenomenological overlap of malnutrition and disability, it would be reasonable to suggest that the two conditions might coexist in some patients undergoing spine surgery. Several bespoke retrospective cohort studies, database inquiries, or prospective research works have already explored the clinical and surgical significance of being malnourished before spine surgery [7,8,9], with malnutrition being often identified as hypoalbuminemia or as an altered body mass index (BMI) [10,11,12]. However, malnourishment is a polyhedric disorder.

Like disability, malnutrition is investigated through dedicated patient-reported questionnaires that screen, for instance, for appetite or involuntary weight changes. Unlike disability, malnutrition in spine surgery is measured through tools that are not condition-specific. The unique signs of malnourishment, like hypophagia, an abnormal body composition, or muscular weakness, require long interviews, sophisticated medical devices, and allocated personnel that examine the patient’s nutritional status. The introduction of nutritional assessment tools is often centre-dependent and they may be not routinely employed. The absence of this information hinders the study of historical data from disease registries. However, it is possible to extrapolate objective information about the patient’s nutritional status from a combination of clinical and laboratory tests to determine a potential diagnosis of malnutrition or a risk of malnutrition. Examples are a difference between the current and ideal body weight of more than 10%, combinations of the lymphocyte count and albumin, and inflammatory markers in the presence of underweight [13].

This retrospective investigation aimed to examine the existence of a relationship between malnutrition and disability in patients undergoing spine surgery. We hypothesised that malnutrition, identified as a combination of multidimensional parameters extracted from routinely collected data, would show an association with the reported degree of disability and physical health. If so, we sought to explore whether malnourished compared to well-fed patients reported higher disability and lower physical scores at admission visits.

## 2. Materials and Methods

### 2.1. Study Design, Setting, and Participants

This investigation was designed as a cross-sectional study involving patients enrolled in the institutional spine disease registry (SpineReg) of the IRCCS Orthopedic Institute Galeazzi. The registry was approved as a prospective research study in September 2015, adding phone interviews before and after surgery to appraise the recovery trends through patient-reported outcome measures. The research was registered in a public trial registry (ClinicalTrials.gov number: NCT03644407; date: 10 November 2015). Any patient waiting for spine surgery at our Italian hospital has been included in SpineReg ever since. The primary data extraction from SpineReg was performed on 19 March 2021 and included all patients with complete records at baseline regarding sex (reported by the doctor as male or female); years of age ≥ 18; a spine diagnosis, excluding those requiring emergency or oncological surgery; the physical status classification system according to the American Society of Anesthesiologists (ASAPS); the ODI; and the summary measure of physical health (PH) from the SF-36. Spine diseases included cervical disorders, complications, deformities (degenerative or idiopathic), disc disease or herniation, spondylolisthesis (degenerative or idiopathic), spondylosis, and stenosis. The calendar time was set for surgeries performed between 2016 and 2019, as, before 2016, the records were less complete, and, after 2020, there were changes to the healthcare systems and population characteristics because of the COVID-19 pandemic. We allowed incomplete records for height, actual body weight (ABW), and BMI. We planned to manually retrieve laboratory results from blood specimens routinely collected at pre-admission to be linked to the primary data extracted from SpineReg. The extraction concerned only the analytes of nutritional interest, as previously described in our technical article [13]: C-reactive protein (CRP, mg·dL^−1^), actual haemoglobin (AHB, g·dL^−1^), mean corpuscular volume (MCV, fL), mean corpuscular haemoglobin (MCH, pg), mean corpuscular haemoglobin concentration (MCHC, g·dL^−1^), neutrophil count (NEUC, 10^3^·μL^−1^), lymphocytic count (LYMC, 10^3^·μL^−1^), prealbumin (PALB, mg·dL^−1^), and albumin (ALB, g·dL^−1^).

### 2.2. Equations to Ascertain the Risk of Malnutrition and Its Diagnosis

Nutritional disorders cover two conditions: undernutrition and overnutrition. In line with this, we applied different calculations that could be performed with our routinely collected parameters to identify patients at risk of undernutrition or overnutrition and those potentially malnourished [13]. Briefly, we calculated the risk of being undernourished or overnourished using the following equations: the body weight difference (BWd) based on the difference between ABW and ideal body weight (IBW); the geriatric nutritional risk index (GNRI), which identifies older patients at risk of undernutrition based on a combination of BWd and circulating ALB; the instant nutritional assessment (INA) derived from ALB and LYMPC; the product of the latter two analytes (LxA); the score of protein malnutrition and acute inflammation (PMA) based on ALB and CRP; the score of protein malnutrition and acute/chronic inflammation (PMAC) based on ALB, PALB, CRP, and the neutrophil–lymphocyte ratio (NLR); the iron deficit malnutrition (IDM) from ABW, the ideal haemoglobin (IHB), and AHB; the vitamin B deficit malnutrition (VBD) based on MCV, MCH, or MCHC. Patients presumably suffering from undernutrition were instead classified using the semi-gold-standard criterion of the Global Leadership Initiative on Malnutrition (GLIM).

### 2.3. Data Extraction and Linkage

The primary SpineReg query obtained data from 3456 patients with eleven variables, encompassing the unique patient code, sex, years of age, metres of height, kilograms of ABW, BMI, orthopaedic condition, ASAPS, year of surgery, ODI, and PH. The manual extraction of laboratory data aimed at collecting nine analytes from the pre-operative routine: CRP, AHB, MCV, MCH, MCHC, NEUC, LYMC, PALB, and ALB. We required no complete records for any of the laboratory data, but at least one record had to be present. Once the linkage was completed, the quality evaluation of the method was verified through the random selection of ten patients per clinical unit per year (a total of 160 patients), followed by a double check of the accuracy of the data from SpineReg and the laboratory parameters. The final database included data from 2258 patients (Table S1). There was poor coverage during the blood linkage process as we eliminated 33.80% of the patients from the primary database. There were no inconsistencies between the primary source (SpineReg) and linked data (laboratory) as only new information was integrated.

### 2.4. Data Cleaning and Coding

The features of the data extracted from the registry and the subsequent categorisation and coding are reported in Table S2. The categorisation process of the ODI and SF-36 scales was based on the quartiles of the cohort, as shown in Table S3, with the quartile method being used for the preliminary assessment of exposure–outcome relationships, rather than for a definitive analysis, because of its disadvantages [14]. The features of the data extracted from the laboratory software system, the reference limits in males and females, and the subsequent coding are reported in Table S4. The nature, categorisation, and coding of the indicators of a malnutritional status are reported in Table S5. The data cleaning phase included the conversion of the measurement units of laboratory analytes if a difference existed regarding the measurement units required by the equations. We also assigned a hard limit of 0.01 mg·dL^−1^ to 45 values of CRP that instead reported <0.02 mg·dL^−1^. All analytes were categorised according to the constraints of reference intervals. The missing values for each variable of the locked database are reported in Table S6. No imputation method or dropping was applied to deal with missing values. A summary of the study is reported in the Supplementary Materials.

### 2.5. Statistics

We planned to report cohort variables as frequencies if categorical or means and standard deviations if continuous. Inferential statistics were performed using the Python programming language (version 3.10.9), by means of the numpy (version 1.21.4), scipy (version 1.10.1), pingouin (version 3.5.2), statsmodels (version 0.13.5), pandas (version 2.0.3), and scikit-learn (version 1.0.2) libraries. Graphics were generated using the matplotlib (version 3.5.2) and seaborn (version 0.12.2) libraries. Sex differences were investigated using the rank-based non-parametric Mann–Whitney U test. The false discovery rate (FDR) due to multiple tests was controlled using the Benjamini–Hochberg FDR procedure [15]. We considered statistical significance at the 95% confidence level (α = 0.05) and effect sizes as rank-biserial correlations (RBCs) (<0.1 = very weak; ≥0.1 and <0.3 = weak; ≥0.3 and <0.5 = moderate; ≥0.5 and <0.7 = strong; ≥0.7 and <1.0 = very strong). The association between malnutrition and disability or physical health was assessed through a two-step procedure designed first to explore the existence of a general association (descriptive analysis) and second to explore the existence of a direction/pattern (analytical analysis). Malnutrition (nine equations) was assumed to be the exposure, and the clinical scores (ODI or SF-36) were considered outcome measures. The descriptive investigation was based on the hypothesis that the categories of malnutrition would show an association with the ODI or PH. One-way analysis of covariance (ANCOVA) based on the ordinary least squares algorithm was used to determine whether there were any statistically significant differences between the outcome means, adjusted for sex, age, and diagnosis, across different levels of malnutrition. Multicollinearity was controlled using the variance inflation factor (VIF), with a dynamic threshold set to the lower bound of the 95% confidence interval for the VIF. The FDR was controlled using the Benjamini–Hochberg FDR procedure. We considered statistical significance at the 95% confidence level (α = 0.05) and effect sizes as the partial eta-squared η^2^ (~0.01 = small; ~0.06 = medium; ~0.14 or higher = large). Assumptions of the one-way ANCOVA were planned to test for appropriateness. The D’Agostino–Pearson omnibus test [16] was used to assess the normality of the residuals, the Goldfeld–Quandt homoscedasticity test [17] verified the presence of homoscedasticity, and the Ljung–Box test verified the absence of autocorrelation of the residuals [18]. The Holm–Bonferroni step-down procedure [19] controlled for the family-wise error rate. We planned to report the adjusted-*p* and η^2^ for all analyses and means ± standard error [95% CI] only for statistically significant associations. The analytical investigation, run only on findings that were found to be statistically significant from the ANCOVA, was based on the assumption that malnourished compared to well-fed patients would report a higher ODI and lower PH. The rank-based non-parametric Jonckheere–Terpstra test for ordered alternatives [20,21] was used to determine if there was a statistically significant monotonic trend between the ordinal malnutrition variable and the ODI or PH. The test was run on the cohorts defined by age group and stratified according to sex and diagnosis, using the Benjamini–Hochberg FDR procedure to control the false discovery rate. Statistical significance was evaluated at the 95% confidence level (α = 0.05) and effect sizes in terms of the r-effect (~0.1 = small; ~0.3 = medium; ~0.5 or higher = large). We planned to report the adjusted-*p* and r-effect with means and the number of observations. Charts were used to report the output from the non-parametric kernel regression technique. The modelling tool was run with the radial basis function kernel support vector regression algorithm [22], where each of the continuous malnutrition scores was an independent variable, the ODI and PH were dependent variables, and age and sex were adjusting covariates. The findings reported in this article adhere to the statement for Reporting Studies Conducted Using Observational Routinely Collected Health Data (RECORD) and were prepared considering the Sex and Gender Equity in Research (SAGER) guidelines.

## 3. Results

The patients’ characteristics are reported in Table 1. The coding of the patients according to the risk of being malnourished or the presumed diagnosis of undernutrition could not be applied to all observations due to missing data (Tables S5 and S6). BWd-derived undernourished patients amounted to 36, while overnourished patients amounted to 672 out of 1041. GNRI categorisation showed 10 individuals with a low risk out of 151.

INA-based categories indicated 172 out of 1336 with energy malnutrition, one with protein malnutrition, and one with protein–energy malnutrition. Through coding with LxA, we found 388 with moderate and 26 with poor nutrition out of 1336. PMA indicated 123 with a high, 92 with a moderate, and 440 with a low malnutritional risk out of 1335. The PMAC percentiles displayed 331 subjects <25th, 331 ≥25th and <50th, 333 ≥50th and <75th, and 334 ≥75th out of 1329. The IDM percentiles showed 259 < 25th, 257 ≥ 25th and <50th, 261 ≥ 50th and <75th, and 260 ≥ 75th out of 1037. The VBD-based labelling of functional vitamin B deficiency identified 452 patients out of 2258. Considering the ascertained diagnosis based on GLIM, the equation could not be applied in 1784 with a high BMI, whereas 44 were identified as potentially suffering from clean undernutrition, 16 from DRM with inflammation, and 95 from DRM without inflammation out of 1939. CRP, MCV, and LYMC were similar between sexes. Non-identical values between females and males were instead found for age (56.31 ± 15.03 vs. 54.98 ± 15.72; *p* = 0.042; 0.05), height (1.62 ± 0.07 vs. 1.75 ± 0.07; *p* < 0.001; 0.84), body weight (65.40 ± 11.71 vs. 80.79 ± 12.28; *p* < 0.001; 0.65), BMI (25.00 ± 4.29 vs. 26.13 ± 3.48; *p* < 0.001; 0.19), AHB (13.40 ± 1.14 vs. 14.94 ± 1.25; *p* < 0.001; 0.67), MCH (29.31 ± 2.20 vs. 29.85 ± 2.24; *p* < 0.001; 0.19), MCHC (32.92 ± 0.97 vs. 33.61 ± 1.04; *p* < 0.001; 0.41), NEUC (4.27 ± 1.80 vs. 4.56 ± 1.89; *p* < 0.001; 0.11), PALB (25.52 ± 4.73 vs. 30.32 ± 5.46; *p* < 0.001; 0.51), and ALB (4.30 ± 0.26 vs. 4.42 ± 0.27; *p* < 0.001; 0.26). Males tended to report lower ODI (39.62 ± 18.06 vs. 48.39 ± 17.16; *p* < 0.001; 0.28) and greater PH (35.23 ± 7.73 vs. 32.69 ± 7.35; *p* < 0.001; 0.20) scores than females. Considering the reference limits as reported in Table S4, we observed high CRP in 201 out of 1251 females and 143 out of 897 males, low AHB in 46 out of 1313 females and 120 out of 945 males, macrocytic MCV in 132 out of 1313 females and 225 out of 945 males, hypochromic MCH in 68 out of 1313 females and 36 out of 945 males, low MCHC in 259 out of 1313 females and 76 out of 945 males, neutropenia in 11 out of 1313 females and 13 out of 945 males, lymphopenia in 35 out of 1313 females and 63 out of 945 males, low PALB in 218 out of 791 females and 31 out of 561 males, and hypoalbuminemia in 2 out of 784 females and 0 out of 552 males.

### 3.1. Younger Adult Patients (<40 Years Old)

The reported levels of disability and physical function among the youngest cohort group, based on malnutrition categorisation, are reported in Table S7. Hereafter, we report the results of the ANCOVA for disability scores based on malnutrition indices: BWd (adjusted-*p* = 0.046; 0.04), INA (adjusted-*p* = 0.283; 0.01), LxA (adjusted-*p* = 0.576; <0.01), PMA (adjusted-*p* = 0.742; 0.01), PMAC (adjusted-*p* = 0.636; 0.02), IDM (adjusted-*p* = 0.475; 0.02), VBD (adjusted-*p* = 0.801; <0.01), and GLIM (adjusted-*p* = 0.713; <0.01). Concerning the PH, the findings were the following: BWd (adjusted-*p* = 0.036; 0.06), INA (adjusted-*p* = 0.283; 0.01), LxA (adjusted-*p* = 0.576; <0.01), PMA (adjusted-*p* = 0.742; 0.01), PMAC (adjusted-*p* = 0.789; 0.01), IDM (adjusted-*p* = 0.475; 0.02), VBD (adjusted-*p* = 0.801; <0.01), and GLIM (adjusted-*p* = 0.573; 0.01). In younger adult patients, only the BWd was statistically significant in terms of both disability (ODI) and the functional status (PH). Younger adult patients identified as having normal nutrition reported an adjusted mean disability score of 32.83 ± 2.08 [28.72–36.95] (*n* = 70), those who were overnourished reported 34.33 ± 2.34 [29.71–38.94] (*n* = 56), and those at risk of being undernourished reported 43.97 ± 4.71 [34.66–53.27] (*n* = 14). The trend of the ODI reported by males was monotonic (adjusted-*p* = 0.048; 0.13): it was 28.24 in well-nourished (*n* = 42), 34.32 in overnourished (*n* = 31), and 55.33 in undernourished (*n* = 3) individuals. Moreover, younger patients admitted with a disc herniation showed a monotonic relationship between the disability scores across the BWd-based malnutrition levels (adjusted-*p* = 0.008; 0.25): 35.00 in well-nourished (*n* = 13), 48.75 in overnourished (*n* = 12), and 48.75 in undernourished (*n* = 4) individuals. The estimated means of physical function were 40.12 ± 0.91 [38.31–41.92] in those with normal nutrition (*n* = 70), 37.97 ± 1.02 [35.94–39.99] in those who were overnourished (*n* = 56), and 35.28 ± 2.06 [31.20–39.36] in those where a risk of undernutrition was identified (*n* = 14).

**Figure 1:**
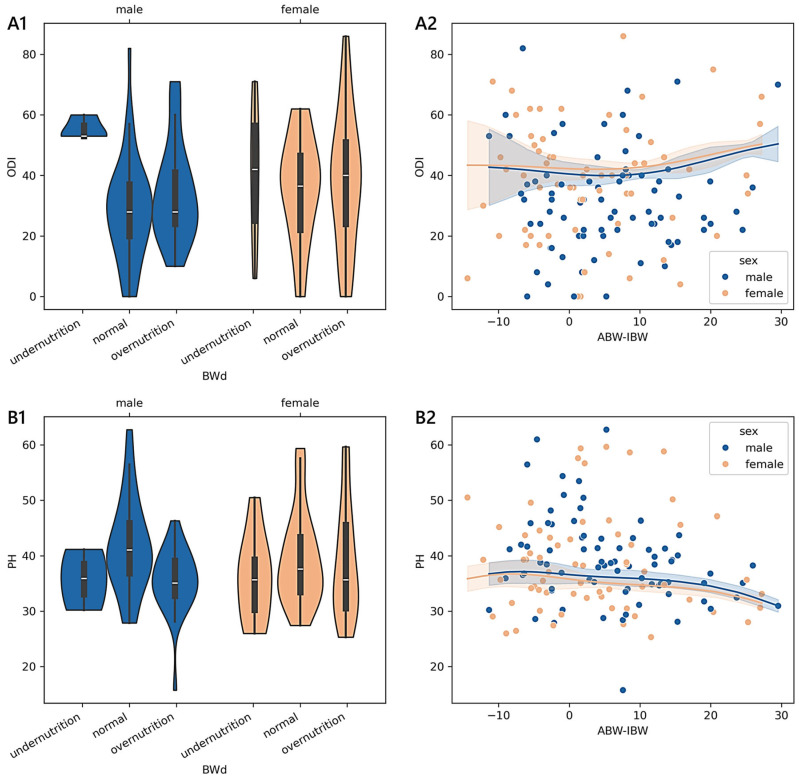
Distributions and trends of outcome measures reported by younger adult patients across degrees of malnutrition. (**A1**,**B1**) show violin plots of the Oswestry disability index (ODI) and physical health (PH) summary measure scores according to different malnutrition levels derived from body weight differences (BWd). White dots are medians, thick bars in the centre of the violins are the interquartile ranges, whiskers represent the rest of the distribution, and violins indicate the shape of the distribution. (**A2**,**B2**) show the non-parametric kernel regression curves of the estimated relationships between the ODI and PH and the difference between the actual body weight (ABW) and ideal body weight (IBW). The lower the number of observations, the wider the bands, which represent the 95% prediction interval around the regressed mean values. Males, blue; females, orange.

The trend of the PH was monotonic in males (adjusted-*p* = 0.023; 0.18): it was 42.64 in well-nourished (*n* = 42), 35.55 in overnourished (*n* = 31), and 35.79 in undernourished (*n* = 3) patients. A pattern of monotonicity was also found for younger patients diagnosed with a complication (adjusted-*p* = 0.012; 0.53; 45.73 in six well-nourished; 34.03 in two overnourished; 39.29 in one undernourished), disc herniation (adjusted-*p* = 0.004; 0.29; 40.03 in 13 well-nourished; 32.06 in 12 overnourished; 35.61 in 4 undernourished), and spondylosis (adjusted-*p* = 0.014; 0.74; 45.71 in three well-nourished; 35.01 in two overnourished). In Figure 1, we report the non-linear relationships between the BWd-based malnutrition and ODI or PH scores for the youngest sample group, adjusted for age and sex. In section A2 of the figure, the values of ABW-IBW associated with the minimum ODI (39.89 in females and 37.28 in males) were 4.28 in females and 5.25 in males, while those associated with the maximum ODI (50.93 in females and 51.34 in males) were 27.22 in females and 29.54 in males. In section B2, the ABW-IBW values that correspond to the minimum PH scores (30.77 in females and 30.87 in males) are 27.22 in females and 29.54 in males, while the maximum PH scores (35.91 in females and 36.58 in males) correspond to −8.10 in females and −6.80 in males.

### 3.2. Adult Patients (40–70 Years Old)

The disability and physical function reported by the subgroup of adults and based on the malnutrition levels are reported in Table S8. The findings from the ANCOVA run with the ODI were the following: BWd (adjusted-*p* = 0.001; 0.02), INA (adjusted-*p* = 0.467; <0.01), LxA (adjusted-*p* = 0.802; <0.01), PMA (adjusted-*p* = 0.006; 0.02), PMAC (adjusted-*p* < 0.001; 0.04), IDM (adjusted-*p* = 0.087; 0.010), VBD (adjusted-*p* = 0.211; 0.001), and GLIM (adjusted-*p* = 0.013; 0.01). The results obtained with the PH scores were the following: BWd (adjusted-*p* = 0.004; 0.02), INA (adjusted-*p* = 0.467; <0.01), LxA (adjusted-*p* = 0.430; <0.01), PMA (adjusted-*p* = 0.007; 0.01), PMAC (adjusted-*p* < 0.001; 0.04), IDM (adjusted-*p* = 0.313; 0.01), VBD (adjusted-*p* = 0.176; <0.01), and GLIM (adjusted-*p* = 0.471; 0.01).

Overall, the ODI and PH reported by adult patients were significantly associated with the categories of BWd, PMA, or PMAC, whereas GLIM was associated only with the ODI. The adjusted disability scores amongst BWd-based labelling were 44.50 ± 1.18 [42.19–46.82] in those with normal nutrition (*n* = 214), 46.36 ± 0.81 [44.76–47.95] in those with overnutrition (*n* = 445), and 58.08 ± 4.02 [50.18–65.98] in those who were potentially undernourished (*n* = 18). No sex- or diagnosis-specific monotonic trend was found. Based on PMA, we observed the following estimated ODI values: 44.04 ± 0.79 [42.48–45.60] (no risk; *n* = 440), 47.24 ± 1.01 [45.26–49.22] (low risk; *n* = 273), 50.35 ± 2.18 [46.07–54.63] (moderate risk; *n* = 58), and 49.18 ± 1.91 [45.44–52.92] (high risk; *n* = 76). The monotonic trend was statistically meaningful for both females (adjusted-*p* = 0.003; 0.12) and males (adjusted-*p* = 0.011; 0.14). We found a statistically meaningful monotonic pattern also for adult patients diagnosed with a complication (adjusted-*p* = 0.002; 0.31) or degenerative deformity (adjusted-*p* = 0.017; 0.26). Among those admitted for a complication, the mean ODI values were 50.76 in those with no risk (*n* = 42), 53.26 in those with a low risk (*n* = 34), 64.38 in those with a moderate risk (*n* = 8), and 67.31 in those with a high risk (*n* = 13). The ODI levels in those with a degenerative deformity were 44.91 in those with no risk (*n* = 35), 46.38 in those with a low risk (*n* = 24), 62.20 in those with a moderate risk (*n* = 5), and 56.43 in those with a high risk (*n* = 7). Similarly, the adjusted disability scores across the PMAC-based groups were 42.33 ± 1.18 [40.07–44.64] (<25th; *n* = 203), 44.05 ± 1.14 [41.81–46.29] (≥25th and <50th; *n* = 210), 47.66 ± 1.11 [45.47–49.84] (≥50th and <75th; *n* = 222), and 49.55 ± 1.15 [47.29–51.81] (≥75th; *n* = 208). The trend was monotonic for both females (adjusted-*p* = 0.021; 0.13) and males (adjusted-*p* = 0.001; 0.24) and for patients with a diagnosis of disc disease (adjusted-*p* = 0.031; 0.22; 43.32 in 50 cases <25th; 41.77 in 56 cases ≥25th and <50th; 49.67 in 51 cases ≥50th and <75th; 50.46 in 46 cases ≥75th), a complication (adjusted-*p* = 0.045; 0.29; 51.92 in 13 cases <25th; 46.95 in 19 cases ≥25th and <50th; 52.33 in 30 cases ≥50th and <75th; 62.74 in 35 cases ≥75th), and a cervical disorder (adjusted-*p* = 0.023; 0.30; 31.97 in 32 cases <25th; 42.48 in 21 cases ≥25th and <50th; 43.05 in 21 cases ≥50th and <75th; 42.46 in 13 cases ≥75th). The adjusted disability levels when using GLIM were 44.69 ± 0.50 [43.71–45.68] (BMI high; *n* = 1179), 46.96 ± 4.47 [38.19–55.72] (clean undernutrition; *n* = 15), 50.67 ± 2.78 [45.21–56.12] (DRM without inflammation; *n* = 39), and 60.16 ± 7.72 [45.02–75.30] (DRM with inflammation; *n* = 5).

There was a monotonic trend for the male sex (adjusted-*p* = 0.016; 0.10; 40.59 in 499 cases with BMI high; 46.50 in two cases with clean undernutrition; 61.25 in four cases with DRM without inflammation; 57.00 in one case with DRM with inflammation) and in patients diagnosed with stenosis (adjusted-*p* = 0.050; 0.17; 43.39 in 87 cases with BMI high; 63.00 in two cases with DRM without inflammation), idiopathic spondylolisthesis (adjusted-*p* = 0.045; 0.24; 38.69 in 48 cases with BMI high; 86.00 in one case with DRM without inflammation), and spondylosis (adjusted-*p* = 0.029; 0.34; 43.59 in 29 cases with BMI high; 59.50 in two cases with DRM without inflammation). Considering the estimated PH, adult patients with BWd-based normal nutrition (*n* = 214) had scores of 34.60 ± 0.48 [33.66–35.54], those who were overnourished (*n* = 445) reported 33.38 ± 0.33 [32.73–34.02], and those with undernutrition (*n* = 18) reported 31.77 ± 1.63 [28.58–34.97]. The trend was monotonic for females (adjusted-*p* = 0.001; 0.11; 34.58 in 111 cases with normal nutrition; 32.31 in 291 cases with overnutrition; 31.09 in 15 cases with undernutrition) and in those with disc disease (adjusted-*p* = 0.004; 0.16; 37.16 in 22 well-nourished; 33.43 in 36 overnourished; 32.77 in two undernourished). The adjusted physical scores based on the PMA-derived risk of malnutrition were 34.31 ± 0.33 [33.67–34.95] (no risk; *n* = 440), 33.03 ± 0.41 [32.22–33.84] (low risk; *n* = 273), 32.28 ± 0.89 [30.52–34.03] (moderate risk; *n* = 58), and 32.51 ± 0.78 [30.97–34.04] (high risk; *n* = 76). The pattern was statistically monotonic for both females (adjusted-*p* = 0.001; 0.12) and males (adjusted-*p* = 0.028; 0.10) and for patients diagnosed with a complication (adjusted-*p* = 0.015; 0.22; 32.92 in 42 cases with no risk; 30.40 in 34 cases with low risk; 28.45 in 8 cases with moderate risk; 30.33 in 13 cases with high risk), a degenerative deformity (adjusted-*p* = 0.017; 0.25; 33.73 in 35 cases with no risk; 32.20 in 24 cases with low risk; 26.73 in 5 cases with moderate risk; 30.42 in 7 cases with high risk), a cervical disorder (adjusted-*p* = 0.009; 0.28; 37.56 in 59 cases with no risk; 36.21 in 19 cases with low risk; 37.00 in 4 cases with moderate risk; 28.08 in 5 cases with high risk), and spondylosis (adjusted-*p* = 0.040; 0.38; 34.58 in 12 cases with no risk; 32.85 in 9 cases with low risk; 27.28 in 3 cases with moderate risk; 29.72 in 5 cases with high risk).

**Figure 2:**
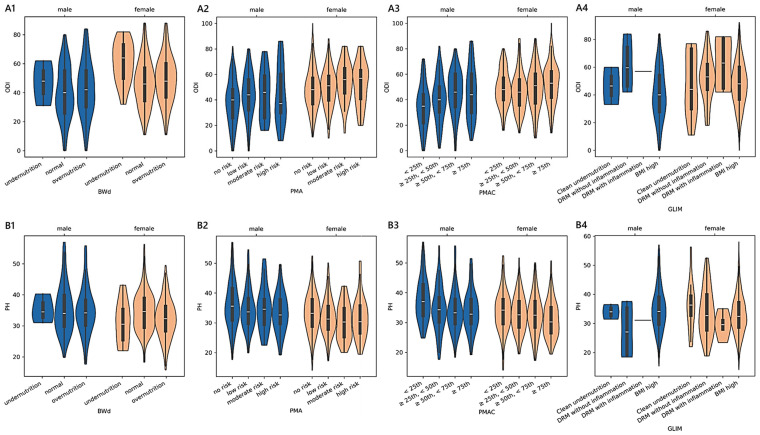
Distributions of outcome measures reported by adult patients across degrees of malnutrition. (**A1**–**A4**) show violin plots of the Oswestry disability index (ODI) and (**B1**–**B4**) of physical health (PH) summary measure scores according to different malnutrition levels derived from body weight differences (BWd), protein malnutrition with acute inflammation (PMA), protein malnutrition with acute and chronic inflammation (PMAC), and the Global Leadership Initiative on Malnutrition (GLIM). White dots are medians, thick bars in the centre of the violins are the interquartile ranges, whiskers represent the rest of the distribution, and violins indicate the shape of the distribution. Males, blue; females, orange.

**Figure 3:**
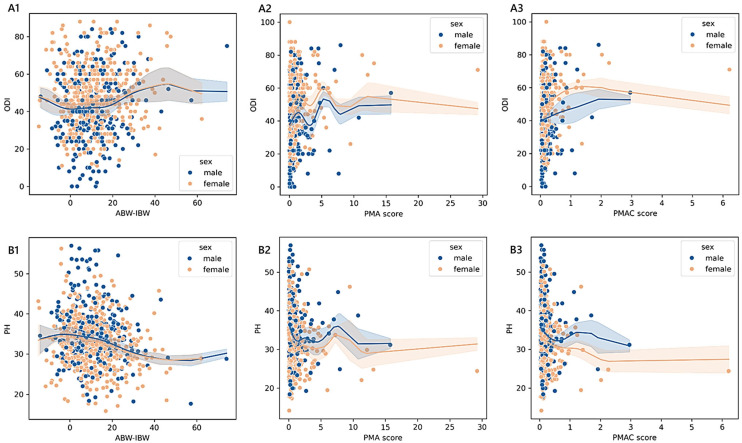
Trends of outcome measures reported by adult patients across degrees of malnutrition. The non-parametric kernel regression curves of the estimated relationships between ODI (**A1**–**A3**) or PH (**B1**–**B3**) and the difference between the actual body weight (ABW) and ideal body weight (IBW), the PMA, and the PMAC. The lower the number of observations, the wider the bands, which represent the 95% prediction interval around the regressed mean values. Males, blue; females, orange.

The PMAC-based estimated physical health scores reported by patients were 35.41 ± 0.48 [34.47–36.35] (<25th; *n* = 203), 33.60 ± 0.47 [32.68–34.51] (≥25th and <50th; *n* = 210), 33.28 ± 0.46 [32.39–34.17] (≥50th and <75th; *n* = 222), and 32.30 ± 0.47 [31.38–33.22] (≥75th; *n* = 208). Similar to PMA, a monotonic trend was also present in females (adjusted-*p* = 0.001; 0.21) and males (adjusted-*p* = 0.001; 0.25), as well as in those admitted for complications (adjusted-*p* = 0.049; 0.24; 32.46 in 13 cases <25th; 33.28 in 19 cases ≥25th and <50th; 31.34 in 30 cases ≥50th and <75th; 29.81 in 35 cases ≥75th), cervical disorders (adjusted-*p* = 0.002; 0.46; 39.55 in 32 cases <25th), 36.49 in 21 cases ≥25th and <50th; 34.77 in 21 cases ≥50th and <75th; 33.13 in 13 cases ≥75th), and spondylosis (adjusted-*p* = 0.042; 0.48; 34.99 in 7 cases <25th; 34.01 in 5 cases ≥25th and <50th; 33.61 in 6 cases ≥50th and <75th; 29.49 in 11 cases ≥75th). The non-linear relationships adjusted for age and sex among BWd, PMA, PMAC, and GLIM and the ODI or PH scores in the subgroup of adult patients are reported in Figure 2 and Figure 3. In section A1 of Figure 3, the scores of ABW-IBW that were associated with the minimum ODI (46.07 in females and 43.98 in males) were −0.91 and 0.72 in females and males, respectively.

The maximum ODI scores (50.83 in females and 50.66 in males) corresponded to 45.73 in females and 46.35 in males. The minimum PH (29.03 in females and 29.16 in males) matched 47.43 in females and 57.20 in males, while −4.19 in females and −2.65 in males matched the maximum PH scores (33.35 in females and 33.10 in males). As shown in section A2, the values of PMA that were associated with the minimum ODI (44.72 in females and 34.55 in males) were 0.02 in females (no nutrition-related risk) and 3.20 in males (high nutrition-related risk). The scores that were associated with the maximum ODI (63.64 in females and 53.27 in males) were 5.37 in females (high nutrition-related risk) and 5.26 in males (high nutrition-related risk). As shown in section B2, the scores of 29.42 in females and 31.98 in males were the minimum PH scores corresponding to the PMA values of 12.15 in females (high nutrition-related risk) and 10.76 in males (high nutrition-related risk), while the maximum PH scores (33.60 in females and 36.59 in males) matched 7.23 in females (high nutrition-related risk) and 0.02 in males (no nutrition-related risk). As shown in section A3, the values of the PMAC scores that were associated with the minimum ODI (47.25 in females and 39.80 in males) were 0.03 in females (<25th) and 0.03 in males (<25th), while those associated with the maximum ODI (56.34 in females and 56.23 in males) were 1.42 in females (≥75th) and 1.92 in males (≥75th). The values of the PMAC scores in section B3 that were associated with the minimum PH scores (30.65 in females and 31.25 in males) were 2.26 in females (≥75th) and 2.96 in males (≥75th), while those that were associated with the maximum PH scores (34.33 in females and 34.59 in males) were 0.03 in females (<25th) and 0.03 in males (<25th).

### 3.3. Older Adult Patients (≥70 Years Old)

In Table S9, we report the disability and physical health reported by older adults, stratified according to different malnutrition codifications. The outputs from the ANCOVA assessing differences in the ODI were as follows: BWd (adjusted-*p* = 0.612; <0.01), GNRI (adjusted-*p* = 0.160; 0.02), INA (adjusted-*p* = 0.509; <0.01), LxA (adjusted-*p* = 0.258; 0.01), PMA (adjusted-*p* = 0.079; 0.02), PMAC (adjusted-*p* = 0.010; 0.04), IDM (adjusted-*p* = 0.005; 0.06), VBD (adjusted-*p* = 0.037; 0.01), GLIM (adjusted-*p* = 0.346; 0.01). The descriptive analysis of PH found the following: BWd (adjusted-*p* = 0.148; 0.02), GNRI (adjusted-*p* = 0.246; 0.01), INA (adjusted-*p* = 0.509; <0.01), LxA (adjusted-*p* = 0.258; 0.01), PMA (adjusted-*p* = 0.079; 0.03), PMAC (adjusted-*p* = 0.009; 0.05), IDM (adjusted-*p* = 0.721; 0.01), VBD (adjusted-*p* = 0.049; 0.01), GLIM (adjusted-*p* = 0.346; 0.01).

In older adults, PMAC, IDM, and VBD were statistically significant in terms of the disability scores, whereas the PH was associated with the PMAC and VBD groups. The estimated disability levels at a greater PMAC-based risk were 45.06 ± 2.40 [40.39–49.72] (<25th; *n* = 45), 46.91 ± 1.87 [43.22–50.60] (≥25th and <50th; *n* = 71), 51.94 ± 1.95 [48.11–55.78] (≥50th and <75th; *n* = 65), and 52.13 ± 1.56 [49.06–55.21] (≥75th; *n* = 101). The pattern was monotonic for females (adjusted-*p* = 0.001; 0.37; 40.00 in 19 cases <25th; 53.17 in 47 cases ≥25th and <50th; 56.45 in 38 cases ≥50th and <75th; 56.35 in 62 cases ≥75th) and for patients with stenosis (adjusted-*p* = 0.018; 0.33; 36.55 in 11 cases <25th; 51.39 in 18 cases ≥25th and <50th; 52.19 in 21 cases ≥50th and<75th; 51.57 in 30 cases ≥75th). The mean adjusted disability scores across IDM categories were 43.81 ± 2.38 [39.11–48.51] (<25th; *n* = 44), 43.95 ± 2.42 [39.18–48.71] (≥25th and <50th; *n* = 45), 51.67 ± 2.02 [47.70–55.65] (≥50th and <75th; *n* = 60), and 52.79 ± 1.93 [48.98–56.60] (≥75th; *n* = 74). Female subjects showed a statistically meaningful, monotonic relationship (adjusted-*p* = 0.001; 0.39; 48.83 in 35 cases <25th; 49.93 in 40 cases ≥25th and <50th; 56.35 in 37 cases ≥50th and <75th; 60.50 in 26 cases ≥75th). When using VBD, patients with an adequate vitamin B status (*n* = 346) had reported estimated disability levels of 48.07 ± 0.85 [46.40–49.75], against 51.05 ± 1.31 [48.48–53.63] in those with a functional deficiency (*n* = 147), with the trend being monotonic for patients diagnosed with a complication (adjusted-*p* = 0.013; 0.37) or degenerative spondylolisthesis (adjusted-*p* = 0.044; 0.20). Among those admitted for a complication, the mean ODI score was 17.87 in 153 cases with an adequate vitamin B status and 64.23 in 13 cases with a functional vitamin B deficiency. Concerning degenerative spondylolisthesis, we found a mean disability score of 44.66 in 70 cases with an adequate vitamin B status, compared to 51.83 in 30 cases with a functional vitamin B deficiency.

**Figure 4:**
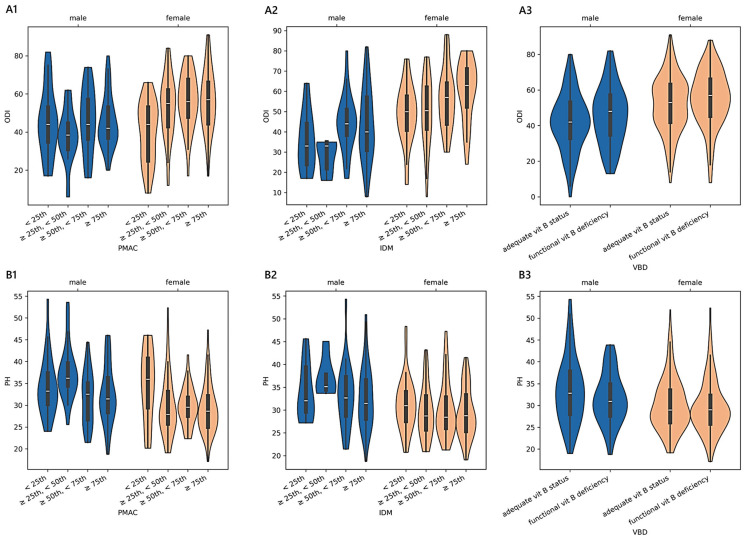
Distributions of outcome measures reported by older adult patients across degrees of malnutrition. (**A1**–**A3**) show violin plots of the Oswestry disability index (ODI) and (**B1**–**B3**) of physical health (PH) summary measure scores according to different malnutrition levels derived from protein malnutrition with acute and chronic inflammation (PMAC), iron deficit malnutrition (IDM), and vitamin B deficit malnutrition (VBD). White dots are medians, thick bars in the centre of the violins are the interquartile ranges, whiskers represent the rest of the distribution, and violins indicate the shape of the distribution. Males, blue; females, orange.

**Figure 5:**
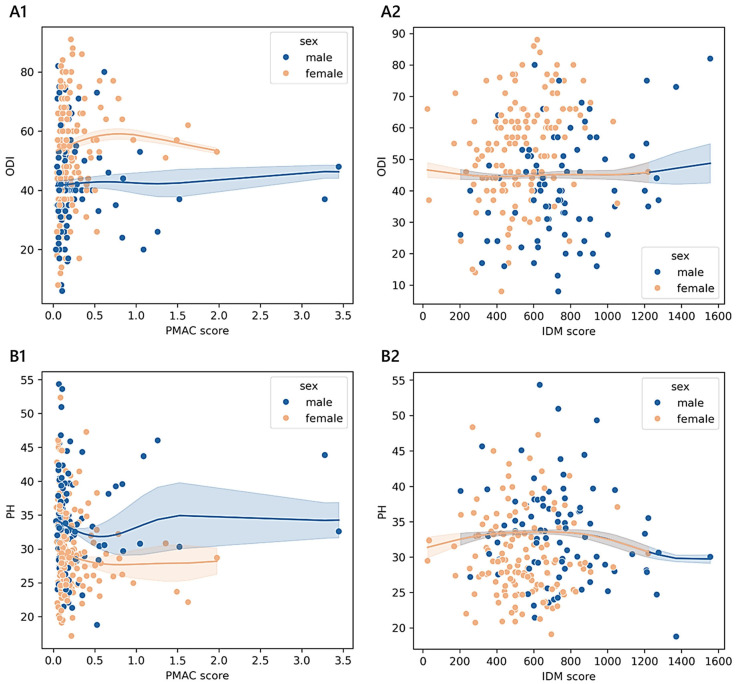
Trends of outcome measures reported by older adult patients across degrees of malnutrition. The non-parametric kernel regression curves of the estimated relationships between the ODI (**A1**,**A2**) or PH (**B1**,**B2**) and PMAC and IDM. The lower the number of observations, the wider the bands, which represent the 95% prediction interval around the regressed mean values. Males, blue; females, orange.

Older adults stratified according to PMAC reported an adjusted mean physical health score of 33.42 ± 0.95 [31.55–35.29] (<25th; *n* = 45), 32.71 ± 0.75 [31.29–34.18] (≥25th and <50th; *n* = 71), 30.61 ± 0.78 [29.07–32.14] (≥50th and <75th; *n* = 65), and 30.47 ± 0.63 [29.23–31.70] (≥75th; *n* = 101). We found a monotonic trend in females (adjusted-*p* = 0.003; 0.30; 34.45 in 19 cases <25th; 29.62 in 47 cases ≥25th and <50th; 29.71 in 38 cases ≥50th and <75th; 29.15 in 62 cases ≥75th) and in patients diagnosed with stenosis (adjusted-*p* = 0.004; 0.45; 35.25 in 11 cases <25th; 29.98 in 18 cases ≥25th and <50th; 29.95 in 21 cases ≥50th and <75th; 28.82 in 30 cases ≥75th). The estimated PH across VBD levels was 31.50 ± 0.35 [30.81–32.18] in patients with an adequate vitamin B status (*n* = 346), while, for those with a functional deficiency (*n* = 147), the mean was 30.47 ± 0.54 [29.42–31.53]. The pattern between the exposure variable and the outcome was monotonic for patients diagnosed with a complication (adjusted-*p* = 0.012; 0.26; 30.98 in 33 cases with adequate vitamin B status and 27.34 in 13 cases with functional vitamin B deficiency). In Figure 4 and Figure 5, we report the relationships between PMAC, IDM, and VBD and the ODI or PH scores for the older adult sample group, adjusted for age and sex.

As shown in section A1 of Figure 5, the values of the PMAC score that corresponded to the minimum ODI (53.25 in females and 40.14 in males) were 1.98 in females (≥75th) and 0.03 in males (<25th), while those matching the maximum ODI (62.17 in females and 48.34 in males) were 0.80 in females (≥25th and <50th) and 3.28 in males (≥75th). As shown in section B1, the PMAC scores that were associated with the minimum PH score (26.06 in females and 31.03 in males) were 0.80 in females (≥25th and <50th) and 0.56 in males (≥75th), while those associated with the maximum PH score (30.71 in females and 33.76 in males) were 0.04 in females (<25th) and 1.52 in males (≥75th). As shown in section A2, the values of IDM that corresponded to the minimum ODI (44.83 in females and 44.81 in males) were 416.48 in females (<25th) and 413.60 in males (<25th), while those associated with the maximum ODI (females: 44.32, males: 40.37) were 25.28 in females (< 25th) and 1556.00 in males (≥75th). As shown in section B2, the values of the IDM score that matched with the minimum PH score (30.44 in females and 29.94 in males) were 1220.48 in females (≥75th) and 1556.00 in males (≥75th), while those associated with the maximum PH score (33.88 in females and 33.88 in males) were 655.76 in females (≥50th and <75th) and 659.84 in males (≥50th and <75th).

## 4. Discussion

In this historical study involving 2258 patients registered in SpineReg between 1 January 2016 and 31 December 2019, aged ≥18 years, who were not undergoing emergency surgery or tumour surgery, we aimed (1) to explore the existence of an association between the attributed malnutritional status and reported disability or physical fitness and (2) to investigate whether there was an ordered pattern in the disability and physical function scores across the different levels of malnutrition at the admission visit before surgery. Malnourished patients were classified according to our previously issued proxy measures of the potential malnutrition risk (BWd, GNRI, INA, LxA, PMA, PMAC, IDM, VDB) and the probable diagnosis (GLIM) [13]. The cohort of the study consisted of 58.15% of female subjects, who tended to be older and shorter and had lower weights. Differences in blood tests reflected the well-known sex differences, with the blood count tending to display higher values in males. Females reported greater levels of disability, by almost nine points on average, and slightly lower scores for PH. These sex differences are not new and could depend on various psychosocial factors and distinct coping mechanisms [23]. Physical function can be influenced by fear avoidance thoughts, being more present in females [24]. Women are also scheduled for surgery when the disease state is more advanced [25]. The overall prevalence of good nourishment was 31.99% when considering BWd, 93.38% for GNRI, 86.98% for INA, 69.01% for LxA, 53.93% for PMA, and 79.98% for VDB. Based on the GLIM, 2.27% had clean undernutrition, 4.90% had DRM without inflammation, and 0.83% had DRM with inflammation; for the remaining 92.01%, it was not possible to calculate the GLIM because the BMI was high. The high variability in these prevalences is due to the constitutive features of the surrogate equations and the inapplicability of some indices (Table S5). Using the classic BMI categorisation, those not having a body mass within the normal ranges were found to constitute 49.58% of the whole study cohort.

The primary findings of this investigation can be summarised as follows.

- Among the 335 younger adults aged less than 40 years, only the BWd equation showed significant differences in terms of the reported disability and functional status while controlling for sex, age, and diagnosis. The predictor variable BWd accounted for approximately 4% of the variance in the ODI and 6% in PH, having a discernible impact in determining how a patient perceived their physical status. We found a clear monotonic trend that was sex- and diagnosis-specific, with the consistency of small effects of malnutrition on the outcome measures being observable only in males. A moderate-to-strong magnitude of the ordered relationship was found concerning disc herniation, complications, and spondylosis. The kernel regression curves illustrated that the least positive difference between ABW and IBW corresponded almost to the lowest disability score and the smallest negative difference corresponded to the highest physical health score (sections A2 and B2 in Figure 1).
- The analysis of the subgroup of 1430 adult patients between 40 and 70 years old revealed that both functional ability scores were statistically significant in terms of malnutrition based on BWd, PMA, and PMAC after adjusting for sex, age, and diagnosis. The practical significance may be limited given the small effect sizes: BWd explained about 2% of the variance in both the ODI and PH, PMA explained 4% in the ODI and 1% in PH, and PMAC explained 4% in both the ODI and PH. Considering BWd, the physical score had an ordered trend only amongst females (small effect) and those with disc disease (small effect), with patterns that revealed higher PH scores in well-nourished compared to undernourished or overnourished patients. Concerning the kernel curves (sections A1 and B1 in Figure 3), we observed the lowest values of the ODI and the highest PH in those who did not deviate too much from the ideal weight. We found a distinguishable monotonic pattern in the ODI and PH across the ordered groups of patients with PMA- and PMAC-derived malnutrition for both sexes (small-to-medium effect) and admission for complications (medium-to-large effect). Significant monotonic patterns in both PROMs were observed in the ordered groups of PMA among those with degenerative deformities (medium effect). Significant patterns in the PH scores were also found across the PMA groups of cervical disorders and spondylosis, as well as among those with disc disease (ODI), cervical disorders (ODI and PH), or spondylosis (PH) concerning PMAC (small-to-medium effect). Overall, as the nutritional risk with PMA or the percentile of PMAC increased, there was a trend towards the worsening of both the disability and physical scores (sections A2, A3, B2, and B3 in Figure 3). Coding based on GLIM revealed a significant association with the ODI across subjects when controlled for sex, age, and diagnosis, despite the small effect size. A statistically significant but moderately strong monotonic trend in the disability scores was observed across the groups of GLIM when considering the male sex or a diagnosis of stenosis, idiopathic spondylolisthesis, or spondylosis. In particular, subjects across the subgroups with a BMI ≥ 20 (not undernourished) or categorised as having clean undernutrition reported reduced disability compared to those with DRM with or without inflammation.
- Considering the subgroup of 493 older adults ≥ 70 years of age, we found significant associations between the ODI and PH based on the PMAC- and VBD-derived malnutrition levels, as well as in the IDM and VBD groups with the ODI alone. Despite the statistical significance, less than 5% of the variance in the outcome measures was accounted for by malnutrition predictors, revealing an overall small effect between the features. However, there was a significant and moderate monotonic trend between the PMAC groups and ODI for females and those with a diagnosis of stenosis, with increasing disability levels reported by those with higher percentiles of PMAC. The categorisation of older patients according to the PMAC equation was similarly able to show a meaningful and substantial pattern with the functional scores in the same subgroups of females and those with an admission for stenosis. The IDM-derived coding showed that the more serious the deficiency, the higher the disability scores reported by the patients. A significant monotonic trend across the groups was observed only for females. Patients labelled with a functional vitamin B deficiency had reported higher levels of disability and lower physical status compared to those with an adequate vitamin B status. The significance of the trend’s monotonicity was diagnosis-specific. Medium effects of malnutrition on both outcomes were observable in those admitted for complications, whereas the degenerative spondylolisthesis subgroup showed a smaller effect only for the disability level. Poor visual interpretability resulted from the kernel regression curves (Figure 5). However, the violin plots in sections A2 and B2 in Figure 4 reasonably demonstrated the greatest ODI and worst PH scores in subjects with the most severe iron deficit malnutrition. The same interpretation can be drawn from sections A3 and B3, where the vitamin B status seems to clearly illustrate different score distributions.

Several parallels can be drawn between our findings and the existing scientific knowledge.

First, the prevalence of BMI-derived malnutrition in our cohort is in line with current evidence showing rates of around 50% [26]. This consistency reinforces the recognition of malnutrition as a common red flag in the pre-operative risk assessment of spine disorders other than spine oncology [27].

Second, we found that small variations in body weight can influence functional outcomes differently across age groups. Specifically, younger adults with a mild weight excess (4–5 kg above IBW) exhibited the lowest disability levels, while those weighing 7–8 kg less than the IBW had the highest physical function scores. Similarly, adults with a slight weight deficit (3–4 kg below IBW) had the highest physical function. These relationships were lost in subjects over 70 years of age. This is not a new discovery as it is known that the body mass in older patients has lower clinical significance compared to the body composition and fat mass [28]. Among older adults with spine pathologies, the back muscular area was associated with the ODI [29]. These findings suggest that a body weight assessment, while providing useful information in adult patients, is not appropriate for older adults, who should instead be assessed regarding their body composition and sarcopenia [30].

Third, the discrimination of functional ability using IDM and VBD, which are predominantly based on red blood cell count analytes, was found to be important only in the geriatric subgroup. Prior research has linked pre-operative disability to markers of iron homeostasis, with the latter being often useful in discriminating patients with iron deficit anaemia [31]. To the authors’ knowledge, the employment of VBD in assuming a certain degree of nutrient deficit is new. The most common vitamin deficiency studied in the orthopaedic field remains hypovitaminosis D, which has been extensively linked to greater disability and worse outcomes after spinal surgery [32]. Our results reinforce the notion that iron deficiency anaemia is a modifiable risk factor in patients undergoing major orthopaedic surgery, together with vitamin D deficiency [33]. More attention is warranted in exploring the role of a nutritional deficiency of B vitamins in aggravating anaemia.

Fourth, we found negligible and non-statistically significant differences in the PROMs when inspecting the GNRI, INA, and LxA categories. This was unexpected as these equations are based on albumin and lymphocytes, which are commonly used as nutritional biomarkers for prognosis and survival after spine surgery [34,35]. The lack of results could be due to the large number of patients missing albumin values (Table S6), which may have reduced the statistical power. However, inconsistent results were also observed by other authors investigating ODI differences among spine patients stratified using a combination of albumin and lymphocyte counts [36]. Notably, PMA and PMAC, which incorporate inflammatory markers like CRP and/or NLR in addition to albumin, were more valid in identifying malnourished patients [37]. This finding suggests that the integration of markers of inflammation into pre-operative nutritional assessment, as a reflection of what could be disease-related malnutrition, might improve risk stratification in spine surgery patients.

### Limitations

First, the malnutritional status was attributed based on surrogate equations. Despite the impossibility of utilising other tools, this method does not reflect the current path in malnutrition screening and diagnosis. Second, the sex analysis stratified by age group elevated our clinical understanding, reduced the noise, and allows generalisation to both sexes in the general population. However, the overall data reliability might be low given that different raters dichotomised the sex and reported PROMs. Third, the involvement of the entire demographic spectrum under the same clinical setting guarantees the repeatability and reproducibility of the findings. Nevertheless, caution should be exercised in generalising these findings beyond the context of our sample. For example, the subgroup of young adults involved only 140 observations, and the small size per group could have reduced the validity. Fourth, the GLIM criteria were designed to diagnose undernutrition (BMI < 20). However, the surrogate equation of the GLIM comprises also the category of BMI ≥ 20. This choice was made to avoid excluding 1179 adults (almost 95.23% of the age subgroup) from the subgroup analysis. Fifth, our investigation did not consider the roles of other clinical, mental, and social components, which could have yielded more accurate results in depicting the precise nature of the relationship. Future research should consider these limitations to produce more influential results that elucidate the primary determinants of disability, physical function, and recovery after surgery.

## 5. Conclusions

In this retrospective study, we have illustrated that the attributed malnutritional status was able to discern differences in self-reported disease-specific functional ability levels among spine patients subjected to elective spine surgery. The consistency of the ordered trends of the relationship between malnutrition and disability or physical fitness depended on age, sex, and the spine pathology. Different malnutrition equations were found to carry varying relevance across age strata: younger adults were most affected by deviations from the ideal body weight, middle-aged patients by inflammation-related protein depletion, and older adults by disruptions in iron homeostasis. Our results encourage researchers to discover methods for historical data manipulation that could help to validate risk prediction models and enable clinical dietitians to incorporate personalised nutrition care as adjunct therapies in prehabilitation. Multidisciplinarity in prehabilitation is essential, but the modalities, clinical value, and sustainability remain to be determined. It is plausible to suggest that interventions will have a greater benefit when applied to more serious cases.

Future efforts should seek to identify clusters of observations and thresholds of features to reasonably establish malnutrition risk-based stratification models of patients and discern those cases that will benefit the most form nutritional prehabilitation.

## Figures and Tables

**Table 1:** The demographic, clinical, surgical, and biochemical characteristics of the study sample.

Variable	Cohort (2258)	Females (1313)			Males (945)		
		Younger (170)	Adults (848)	Older (295)	Younger (165)	Adults (582)	Older (198)
Age, y	55.76 ± 15.33 (2258)	29.32 ± 6.80 (170)	55.15 ± 8.00 (848)	75.21 ± 3.68 (295)	30.70 ± 6.57 (165)	54.96 ± 8.73 (582)	75.29 ± 3.89 (198)
mH, m	1.67 ± 0.10 (1041)	1.64 ± 0.06 (64)	1.62 ± 0.07 (417)	1.59 ± 0.06 (139)	1.77 ± 0.07 (76)	1.75 ± 0.07 (260)	1.73 ± 0.06 (85)
ABW, kg	71.62 ± 14.13 (1041)	58.88 ± 11.58 (64)	66.25 ± 11.85 (417)	65.85 ± 10.41 (139)	77.91 ± 10.87 (76)	81.80 ± 12.29 (260)	80.28 ± 13.14 (85)
BMI, kg·(m^2^)^−1^	25.47 ± 4.01 (1939)	22.26 ± 3.82 (145)	25.17 ± 4.25 (732)	26.08 ± 4.03 (246)	24.89 ± 3.14 (143)	26.30 ± 3.52 (506)	26.66 ± 3.42 (167)
CRP, mg/dL	0.35 ± 0.74 (2148)	0.23 ± 0.42 (162)	0.35 ± 0.69 (809)	0.48 ± 1.09 (280)	0.18 ± 0.31 (159)	0.33 ± 0.60 (551)	0.48 ± 1.08 (187)
AHB, g/dL	14.04 ± 1.40 (2258)	13.29 ± 1.06 (170)	13.47 ± 1.12 (848)	13.27 ± 1.20 (295)	15.35 ± 1.02 (165)	15.02 ± 1.18 (582)	14.34 ± 1.40 (198)
MCH, pg	29.53 ± 2.23 (2258)	29.05 ± 2.37 (170)	29.39 ± 2.18 (848)	29.23 ± 2.14 (295)	29.58 ± 1.61 (165)	29.80 ± 2.45 (582)	30.20 ± 2.02 (198)
MCHC, g/dL	33.21 ± 1.05 (2258)	33.11 ± 0.96 (170)	32.96 ± 0.96 (848)	32.67 ± 0.97 (295)	33.93 ± 0.94 (165)	33.61 ± 1.04 (582)	33.33 ± 1.03 (198)
MCV, fL	88.90 ± 5.75 (2258)	87.67 ± 6.19 (170)	89.11 ± 5.57 (848)	89.42 ± 5.74 (295)	87.18 ± 4.19 (165)	88.62 ± 6.25 (582)	90.58 ± 5.06 (198)
NEUC, 10^3^/μL	4.39 ± 1.84 (2258)	4.31 ± 1.93 (170)	4.21 ± 1.78 (848)	4.42 ± 1.76 (295)	4.19 ± 1.58 (165)	4.59 ± 1.90 (582)	4.77 ± 2.04 (198)
LYMC, 10^3^/μL	2.25 ± 0.79 (2258)	2.41 ± 0.74 (170)	2.24 ± 0.82 (848)	2.11 ± 0.81 (295)	2.42 ± 0.69 (165)	2.30 ± 0.72 (582)	2.05 ± 0.89 (198)
PALB, mg/dL,	27.51 ± 5.57 (1352)	25.28 ± 4.94 (105)	25.61 ± 4.72 (517)	25.38 ± 4.64 (169)	31.02 ± 5.30 (101)	30.74 ± 5.47 (341)	28.52 ± 5.22 (119)
ALB, g/dL	4.35 ± 0.27 (1336)	4.39 ± 0.26 (104)	4.30 ± 0.25 (513)	4.22 ± 0.25 (167)	4.61 ± 0.24 (101)	4.41 ± 0.25 (335)	4.27 ± 0.25 (116)
ODI	44.72 ± 18.06 (2258)	39.85 ± 20.04 (170)	48.51 ± 16.35 (848)	52.98 ± 15.78 (295)	32.38 ± 17.40 (165)	40.53 ± 18.35 (582)	42.97 ± 16.14 (198)
PH	33.75 ± 7.62 (2258)	36.99 ± 9.12 (170)	32.76 ± 6.94 (848)	30.02 ± 6.06 (295)	39.18 ± 8.12 (165)	34.90 ± 7.40 (582)	32.94 ± 7.18 (198)
Cervical disorder	10.76 (243)	5.29% (9)	11.20% (95)	6.78% (20)	9.70% (16)	13.75% (80)	11.62% (23)
Complication	9.70 (219)	8.82% (15)	12.26% (104)	11.86% (35)	4.85% (8)	7.90% (46)	5.56% (11)
Deformity, degenerative	9.48 (214)	0% (0)	14.39% (122)	16.27% (48)	0% (0)	4.64% (27)	8.59% (17)
Deformity, idiopathic	5.58 (126)	30.00% (51)	3.89% (33)	0% (0)	16.97% (28)	2.41% (14)	0% (0)
Disc disease	19.09 (431)	14.71% (25)	21.23% (180)	14.92% (44)	15.15% (25)	22.68% (132)	12.63% (25)
Disc herniation	16.43 (371)	22.35% (38)	11.67% (99)	4.41% (13)	35.76% (59)	24.40% (142)	10.10% (20)
Spondylolisthesis, degenerative	11.29 (255)	0% (0)	13.09% (111)	21.02% (62)	0% (0)	7.56% (44)	19.19% (38)
Spondylolisthesis, idiopathic	4.61 (104)	14.71% (25)	4.25% (36)	0% (0)	13.94% (23)	3.44% (20)	0% (0)
Spondylosis	2.66 (60)	4.12% (7)	2.36% (20)	2.37% (7)	2.42% (4)	2.58% (15)	3.54% (7)
Stenosis	10.41 (235)	0% (0)	5.66% (48)	22.37% (66)	1.21% (2)	10.65% (62)	28.79% (57)

Notes: Continuous variables are reported as the mean ± standard deviation (number of cases) and categorical variables are reported as frequencies (number of cases). Younger adults are <40 years old, adults 40–70 years old, and older adults ≥ 70 years old. Abbreviations: mH, metres of height; ABW, actual body weight; BMI, body mass index; ASAPS, American Society of Anesthesiologists’ Classification of Physical Status; ODI, Oswestry disability index; PH, physical health summary measure; CRP, C-reactive protein; AHB, actual haemoglobin; MCV, mean corpuscular volume; MCH, mean corpuscular haemoglobin; MCHC, mean corpuscular haemoglobin concentration; NEUC, neutrophil count; LYMC, lymphocyte count; PALB, prealbumin; ALB, albumin.

## Data Availability

The data analysed in this article are shared, after deidentification, immediately and indefinitely as supplementary raw data with the publication, together with the Supplementary Materials, RECORD checklist, and SAGER checklist.

## Supplementary Materials

The following supporting information can be downloaded at: https://www.mdpi.com/article/10.3390/medicina61030413/s1, Figure S1: Summary slide; Table S1: Number of patients in the linkage process between the institutional spine registry (SpineReg) and blood analytes; Table S2: Clinical and surgical data extracted from the registry; Table S3: The categorization of the ODI and SF-36 physical score of the study cohort into quartiles; Table S4: Laboratory parameters, relative normal ranges, and categorization; Table S5: Indices of malnutrition from a combination of clinical and laboratory tests: Table S6: Missing values; Table S7: The malnutritional status, disability, and functional status in the 335 younger adult patients; Table S8: The malnutritional status, disability, and functional status in the 1430 adult patients; Table S9: The malnutritional status, disability, and functional status in the 493 older adults.

## Abbreviations

The following abbreviations are used in this manuscript:

ABW	actual body weight
AHB	actual haemoglobin
ALB	albumin
ANCOVA	analysis of covariance
ASAPS	American society of Anesthesiologists’ Classification of Physical Status
BMI	body mass index
BWd	body weight difference
CRP	C-reactive protein
DRM	disease-related malnutrition
FDR	false discovery rate
GLIM	Global Leadership Initiative on Malnutrition
GNRI	geriatric nutritional risk index
IBW	ideal body weight
IDM	iron deficit malnutrition
HB	ideal haemoglobin
INA	instant nutritional assessment
LxA	combination of lymphocyte count and albumin
LYMC	lymphocyte count
MCH	mean corpuscular haemoglobin
MCHC	mean corpuscular haemoglobin concentration
MCV	mean corpuscular volume
mH	metres of height
NEUC	neutrophil count
NLR	neutrophil–lymphocyte ratio
ODI	Oswestry disability index
PALB	prealbumin
PH	physical health summary measure
PMA	protein malnutrition with acute inflammation
PMAC	protein malnutrition with acute and chronic inflammation
PROMs	patient-reported outcome measures
SF-36	36-item Short-Form Health Survey
upc	unique patient code
VBM	vitamin B deficit malnutrition
VIF	variance inflation factor
YoS	year of surgery
