# Supplementary material for: Malnutrition and Disability: A Retrospective Study on 2258 Adult Patients Undergoing Elective Spine Surgery
Source: Medicina (Kaunas). 2025 Feb 26;61(3):413. doi: 10.3390/medicina61030413 (PMC11943876; doi:10.3390/medicina61030413)
Supplement: Supplementary file 1 [file medicina-61-00413-s001.zip › Supplementary_Files/EASE_SAGER_Checklist.pdf]

# Table 1. SAGER guidelines checklist
# Studies with human participants

| Section / topic | Item number | Checklist item | Reported on page number |
|---|---|---|---|
| General | | | |
| | 1 | The terms sex/gender used appropriately | reported |
| Title | | | |
| | 2 | Title specifies the sex/gender of participants if only one included | both included |
| Abstract | | | |
| | 3a | Abstract specifies the sex/gender of participants if only one included | both included |
| | 3b | Study population described with sex/gender breakdown* | described |
| Introduction | | | |
| | 4a | If relevant, previous studies that show presence or lack of sex/gender differences or similarities are cited | not discussed |
| | 4b | Mention of whether sex/gender might be an important variant and if differences might be expected | not mentioned in the introduction |
| | 4c | The demographics of the study population with regard to sex/gender (eg, disease prevalence among male/female study participants) are outlined* | not mentioned in the introduction |
| Methods | | | |
| | 5a | Method of definition of sex/gender (eg, self-report, genetic testing) | reported |
| | 5b | Description of how sex/gender was considered in the design, whether authors ensured adequate representation of male and female study participants, justification of the reasons for any exclusion of male or female participants, or explanation if not considered. Justification of other sex/gender-specific interventions of study designs (eg, mandating contraception for women).* Explicit reporting of the scientific rationale for | described |

| | | | |
|---|---|---|---|
| | | contraception requirements and exclusions for pregnancy and lactation should be required* | not reported |
| Results | | | |
| | 6a | Study population description with complete gender/sex breakdown for all categories considered* | Tables |
| | 6b | Where appropriate, data presented disaggregated by sex/gender, and sex/gender differences and similarities are described | Tables |
| | 6c | Sex- and gender-based analyses reported regardless of outcome (in main paper if pre-specified; otherwise in appendix)* | Tables |
| | 6d | For clinical trials, adverse event data disaggregated by sex/gender (in main paper if pre-specified; otherwise in appendix)* | not applicable |
| | 6e | Patient-reported outcome data disaggregated by sex/gender (in main paper if pre-specified; otherwise in appendix)* | Tables |
| | 6f | For epidemiological studies, the effects of other exposures on health problems examined for all genders and analysed critically from a gender perspective | not discussed |
| | 6g | Table 1 includes separate rows for male sex/gender, female sex/gender and other categories if collected* | Table 1 |
| Discussion | | | |
| | 7a | Potential implications of sex/gender on the study results and analyses, including the extent to which the findings can be generalized to all sexes/genders in a population | reported |
| | 7b | If a sex/gender analysis not done, a rationale is given and implications of the lack of such analysis on the interpretation of the results are discussed | not applicable |

Adapted from SAGER guidelines. Sex and Gender Equity in Research: rationale for the SAGER guidelines and recommended use. Research Integrity and Peer Review 1, Article number: 2 (2016) https://researchintegrityjournal.biomedcentral.com/articles/10.1186/s41073-016-0007-6.
* These points extend beyond the original SAGER table
