# Supplementary material for: Malnutrition and Disability: A Retrospective Study on 2258 Adult Patients Undergoing Elective Spine Surgery
Source: Medicina (Kaunas). 2025 Feb 26;61(3):413. doi: 10.3390/medicina61030413 (PMC11943876; doi:10.3390/medicina61030413)
Supplement: Supplementary file 1 [file medicina-61-00413-s001.zip › Supplementary_Files/Supplementary_Tables.pdf]

medicina

MDPI

Supplementary tables

Table S1. Number of patients in the linkage process between the institutional spine registry (SpineReg) and blood analytes.

SpineReg (year)	Prime extraction	Duplicate removal	Blood linkage
2016	748	739	489
2017	967	963	616
2018	640	628	380
2019	1101	1081	773
total	3456	3411	2258

Table S2. Clinical and surgical data extracted from the registry.

Variable	Nature	Measure	Categories and coding
upc	discrete	none	none
sex	nominal, male OR female	none	male=0; female=1
age	continuous	years	<40 years (younger adults)=0; 40–70 years (adults)=1; ≥70 years (older adults)=2
mH	continuous	metres	None
ABW	continuous	kilograms	None
BMI	continuous	$kg \cdot (m^2)^{-1}$	if younger adults and adults: <18.5 (underweight)=0; 18.5–24.9 (normal)=1; 25.0–29.9 (overweight)=2; 30.0–34.9 (obesity I)=3; 35.0–39.9 (obesity II)=4; ≥40.0 (obesity III)=5.<br>if seniors[(a)]: <25.0 (underweight)=0; 25.0–35.0 (normal)=1; 35.1–40.0 (overweight)=2; 40.1–45.0 (obesity I)=3; 45.1–50.0 (obesity II)=4; ≥50.1 (obesity III)=5.
diagnosis	nominal, 10 disorders	none	orthopedic conditions: cervical disorder=0; complication=1; deformity (degenerative)=2; deformity (idiopathic)=3; disc disease=4; disc herniation=5; spondylolisthesis (degenerative)=6; spondylolisthesis (idiopathic)=7; spondylosis=8; stenosis=9
ASAPS	nominal, ordinal	none	healthy=1; mild disease=2; severe disease=3; threat to life=4; moribund=5; brain-dead=6
YoS	discrete	none	2016=0; 2017=1; 2018=2; 2019=3

Notes: [(a)]The Quetelet index (Nuttall 2015) has been adjusted for advanced age (Kıskaç *et al.* 2022). **Abbreviations:** upc, unique patient code; mH, metres of height; ABW, actual body weight; BMI, body mass index; ASAPS, American society of anesthesiologists' classification of physical status; YoS, year of surgery.

 https://doi.org/10.3390/medicina61030413

Table S3. The categorization of the ODI and SF-36 physical score of the study cohort into quartiles.

Variable	Categorization				Significance of the dimensions investigated	Scoring system
	Q1	Q2	Q3	Q4		
ODI	<32.00	<44.00	<57.00	≥57.00	disease-specific disability status	0 (best) – 100 (worst)
PH	<28.44	<32.98	<38.14	≥38.14	general QoL from physical status during the last 4 weeks	0 (worst) – 100 (best)

Notes: The four categories derived from quartiles are: <25th percentile, ≥25th percentile and <50th percentile, ≥50th percentile and <75th percentile; ≥75th percentile. **Abbreviations:** ODI, Oswestry disability index; SF-36, 36-item short form health survey; PH, the summary measure of physical health.

Table S4. Laboratory parameters, relative normal ranges, and categorization.

Indicator	Unit of measure	Reference limits in males		Reference limits in females		Categories and coding notes
		lower	upper	lower	upper	
CRP	$mg \cdot dL^{-1}$	0.00	0.50	0.00	0.50	normal=0; high=1
AHB	$g \cdot dL^{-1}$	13.70	17.50	11.20	15.70	low=0; normal=1; high=2
MCV	fL	79.00	92.20	79.40	94.80	microcytic=0; eucytic=1; macrocytic=2
MCH	pg	25.70	32.20	25.60	32.20	hypochromic=0; euchromic=1; hyperchromic=2
MCHC	$g \cdot dL^{-1}$	32.30	36.50	32.20	35.50	low=0; normal=1; high=2
NEUC	$10^3 \cdot \mu L^{-1}$	1.78	5.38	1.56	6.13	neutropenia=0; normal=1; neutrocytosis=2
LYMC	$10^3 \cdot \mu L^{-1}$	1.32	3.57	1.18	3.74	lymphopenia=0; normal=1; lymphocytosis=2
PALB	$mg \cdot dL^{-1}$	23.00	42.00	23.00	40.00	low=0; normal=1; high=2
ALB	$g \cdot dL^{-1}$	3.50	5.20	3.50	5.20	hypoalbuminemia=0; eualbuminemia=1; hyperalbuminemia=2

Abbreviations: CRP, C-reactive protein; AHB, actual haemoglobin; MCV, mean corpuscular volume; MCH, mean corpuscular haemoglobin; MCHC, mean corpuscular haemoglobin concentration; NEUC, neutrophils count; LYMC, lymphocytes count; PALB, prealbumin; ALB, albumin.

Table S5. Indices of malnutrition from a combination of clinical and laboratory tests.

Variable	Nature	Measure	Categories and coding	Non applicable in
BWd	continuous	kilograms	If the absolute difference between ABW and IBW within 10% IBW=1 (normal) If over 10% IBW=0 (undernutrition) or 2 (overnutrition)	1217 subjects with no ABW.
GNRI	continuous	none	Literature-based categories of nutrition-related risk are: absent=0; low=1; moderate=2; major=3	1765 younger adults or adults; 210 seniors with no ALB; 132 seniors with no ABW.
INA	nominal	none	Literature-based categories are: well-nourished=0; protein malnutrition=1; energy malnutrition=2; protein-energy malnutrition=3	922 subjects with no ALB.
LxA	continuous	none	Literature-based categories are: poor=0; middle=1; good=2	922 subjects with no ALB.
PMA	continuous	none	Literature-based categories are: no risk=0; low risk=1; moderate risk=2; high risk=3	922 subjects with no ALB; 1 subject with no CRP.
PMAC	continuous	none	Cohort-based percentiles are:[(a)] <25th=0; ≥25th and <50th=1; ≥50th and <75th=2; ≥75th=3	929 subjects with no NLR, CRP, ALB, or PALB.
IDM[(b)]	continuous	mg	Cohort-based percentiles:[(c)] <25th=0; ≥25th and <50th=1; ≥50th and <75th=2; ≥75th=3	1217 subjects with no ABW; 4 subjects with IHB lower than AHB.
VBD	nominal, ordinal	none	Literature-based categories are: adequate vit. B status=0; functional vit. B deficiency=1	0 subjects.
GLIM	nominal, ordinal	none	Literature-based categories are: clean undernutrition=1; DRM without inflammation=2; DRM with inflammation=3; BMI high=0	1784 subjects with BMI ≥ 20 (adults) or ≥ 22 (seniors); 319 subjects with no BMI.

Notes: NLR is used as a measure of systemic inflammation in the GLIM and PMAC: <2 is normal (coded 0), 2-3.99 is low (coded 1); 4-5.99 is mild (coded 2); 6-7.99 is moderate (coded 3); ≥8 is severe (coded 4). [(a)]Percentiles of the PMAC are: Q1 = 0.08; Q2 = 0.12; Q3 = 0.19. [(b)]IHB is the mean value of the normal reference range: 15.6 $g \cdot dL^{-1}$ for males and 13.5 $g \cdot dL^{-1}$ for females. [(c)]Percentiles of the IDM are: Q1 = 424.4; Q2 = 538.4; Q3 = 679.3. **Abbreviations:** BWd, body weight difference; ABW, actual body weight; IBW, ideal body weight; GNRI, geriatric nutritional risk index; ALB, albumin;; INA, instant nutritional assessment; LxA, combination of lymphocyte count and albumin; PMA, protein malnutrition with acute inflammation; CRP, C-reactive protein; PMAC, protein malnutrition with acute and chronic inflammation; NLR, neutrophil-lymphocyte ration; PALB, prealbumin; IDM, iron deficit malnutrition; IHB, ideal haemoglobin; AHB, actual haemoglobin; VBM, vitamin B deficit malnutrition; GLIM, global leadership initiative on malnutrition; DRM, disease-related malnutrition; BMI, body mass index.

Table S6. Missing values.

Source of data	Acronym	Total (n)	Missing (n)	Missing (%)
SpineReg	Sex	2258	0	0
	Age	2258	0	0
	mH	1041	1217	53.90%
	ABW	1041	1217	53.90%
	BMI	1939	319	14.13%
	Diagnosis	2258	0	0
	ASAPS	2258	0	0
	YoS	2258	0	0
	ODI	2258	0	0
	PH	2258	0	0
Blood analytes	CRP	2148	110	4.87%
	AHB	2258	0	0
	MCV	2258	0	0
	MCH	2258	0	0
	MCHC	2258	0	0
	NEUC	2258	0	0
	LYMPC	2258	0	0
	PALB	1352	906	40.12%
	ALB	1336	922	40.83%
Imputations	BWd	1041	1217	53.90%
	GNRI	151	342	69.37%
	NLR	2258	0	0
	INA	1336	922	40.83%
	LxA	1336	922	40.83%
	PMA	1335	923	40.88%
	PMAC	1329	929	41.14%
	IDM	1037	1217	53.99%
	VBM	2258	0	0
	GLIM	1939	319	14.13%

Abbreviations: mH, metres of height; ABW, actual body weight; BMI, body mass index; ASAPS, American society of anesthesiologists’ classification of physical status; YoS, year of surgery; ODI, Oswestry disability index; PH, physical health summary measure; CRP, C-reactive protein; AHB, actual haemoglobin; MCV, mean corpuscular volume; MCH, mean corpuscular haemoglobin; MCHC, mean corpuscular haemoglobin concentration; NEUC, neutrophils count; LYMC, lymphocytes count; PALB, prealbumin; ALB, albumin; BWd, body weight difference; GNRI, geriatric nutritional risk index; NLR, neutrophil-lymphocyte ration; INA, instant nutritional assessment; LxA, combination of lymphocyte count and albumin; PMA, protein malnutrition with acute inflammation; PMAC, protein malnutrition with acute and chronic inflammation; IDM, iron deficit malnutrition; VBM, vitamin B deficit malnutrition; GLIM, global leadership initiative on malnutrition.

Table S7. The malnutritional status, disability, and functional status in the 335 younger adult patients.

		**ODI**		**PH**	
		Female (170)	Male (165)	Female (170)	Male (165)
BWd	undernutrition	41.18 ± 12.57 (11)	55.33 ± 4.57 (3)	36.04 ± 4.54 (11)	35.79 ± 6.20 (3)
	normal	35.96 ± 6.46 (28)	28.24 ± 5.04 (42)	39.45 ± 3.12 (28)	41.64 ± 2.53 (42)
	overnutrition	38.40 ± 8.69 (25)	34.32 ± 5.79 (31)	38.74 ± 3.99 (25)	35.55 ± 2.02 (31)
INA	well-nourished	38.52 ± 4.21 (93)	32.44 ± 3.63 (93)	37.55 ± 1.89 (93)	39.44 ± 1.58 (93)
	protein malnutrition	null (0)	null (0)	null (0)	null (0)
	energy malnutrition	32.00 ± 13.81 (11)	27.88 ± 13.39 (8)	40.46 ± 6.42 (11)	40.66 ± 6.56 (8)
	protein-energy malnutrition	null (0)	null (0)	null (0)	null (0)
LxA	poor nutrition	null (0)	null (0)	null (0)	null (0)
	middle nutrition	35.62 ± 7.85 (26)	30.50 ± 8.41 (16)	38.33 ± 3.36 (26)	40.60 ± 5.04 (16)
	good nutrition	38.56 ± 4.71 (78)	32.38 ± 3.85 (85)	37.70 ± 2.16 (78)	39.34 ± 1.57 (85)
PMA	no risk of malnutrition	36.38 ± 4.77 (68)	32.17 ± 3.94 (75)	38.13 ± 2.14 (68)	38.98 ± 1.60 (75)
	low risk of malnutrition	39.23 ± 8.27 (26)	31.74 ± 9.51 (19)	37.78 ± 3.70 (26)	42.16 ± 4.17 (19)
	moderate risk of malnutrition	45.50 ± 18.74 (4)	35.50 ± 16.35 (4)	32.05 ± 8.87 (4)	36.11 ± 7.71 (4)
	high risk of malnutrition	43.00 ± 25.38 (6)	27.33 ± 19.77 (3)	39.00 ± 11.75 (6)	41.47 ± 19.31 (3)
PMAC	< 25th	32.84 ± 6.50 (32)	31.31 ± 4.38 (51)	38.56 ± 3.27 (32)	39.97 ± 2.02 (51)
	≥ 25th, < 50th	39.46 ± 8.59 (24)	33.27 ± 7.61 (26)	38.24 ± 3.89 (24)	37.98 ± 2.32 (26)
	≥ 50th, < 75th	39.12 ± 6.70 (33)	34.31 ± 10.90 (13)	37.72 ± 2.63 (33)	40.48 ± 5.39 (13)
	≥ 75th	43.00 ± 13.62 (15)	32.10 ± 13.29 (10)	36.05 ± 6.51 (15)	39.51 ± 7.41 (10)
IDM	< 25th	38.89 ± 7.58 (19)	27.68 ± 7.80 (19)	36.82 ± 3.86 (19)	38.13 ± 3.91 (19)
	≥ 25th, < 50th	40.05 ± 9.01 (20)	36.16 ± 7.42 (19)	38.81 ± 4.00 (20)	39.06 ± 3.91 (19)
	≥ 50th, < 75th	38.31 ± 10.69 (16)	31.40 ± 8.04 (15)	38.72 ± 4.62 (16)	37.16 ± 2.52 (15)
	≥ 75th	29.67 ± 14.17 (9)	31.83 ± 7.49 (23)	41.58 ± 6.14 (9)	40.63 ± 3.26 (23)
VBD	adequate vit. B status	40.06 ± 3.18 (143)	32.37 ± 2.72 (154)	36.74 ± 1.50 (143)	39.16 ± 1.28 (154)
	functional vit. B deficiency	38.74 ± 8.87 (27)	32.45 ± 12.44 (11)	38.32 ± 3.42 (27)	39.38 ± 5.32 (11)
GLIM	clean undernutrition	36.96 ± 8.35 (23)	17.33 ± 7.28 (3)	39.02 ± 3.78 (23)	53.19 ± 5.17 (3)
	DRM without inflammation	34.94 ± 9.94 (16)	12.00 ± 7.84 (2)	39.44 ± 5.46 (16)	46.76 ± 15.43 (2)
	DRM with inflammation	43.67 ± 26.74 (3)	null (0)	37.94 ± 9.27 (3)	null (0)
	BMI high	40.97 ± 3.86 (103)	33.20 ± 2.92 (138)	36.24 ± 1.72 (103)	38.75 ± 1.31 (138)

Notes: Variables are reported as mean ± standard deviation (number of cases). **Abbreviations:** mH, metres of height; ABW, actual body weight; BMI, body mass index; ASAPS, American society of anesthesiologists' classification of physical status; ODI, Oswestry disability index; PH, physical health summary measure; CRP, C-reactive protein; AHB, actual haemoglobin; MCV, mean corpuscular volume; MCH, mean corpuscular haemoglobin; MCHC, mean corpuscular haemoglobin concentration; NEUC, neutrophils count; LYMC, lymphocytes count; PALB, prealbumin; ALB, albumin.

Table S8. The malnutritional status, disability, and functional status in the 1430 adult patients.

		**ODI**		**PH**	
		Female (848)	Male (582)	Female (848)	Male (582)
BWd	undernutrition	61.07 ± 7.22 (15)	47.00 ± 17.57 (3)	31.09 ± 3.45 (15)	35.28 ± 5.32 (3)
	normal	46.68 ± 2.99 (111)	40.00 ± 3.52 (103)	34.58 ± 1.19 (111)	35.47 ± 1.52 (103)
	overnutrition	49.10 ± 1.83 (291)	42.55 ± 2.98 (154)	32.31 ± 0.72 (291)	34.82 ± 1.20 (154)
INA	well-nourished	48.92 ± 1.40 (455)	40.97 ± 2.06 (296)	32.70 ± 0.60 (455)	35.16 ± 0.83 (296)
	protein malnutrition	68.00 (1)	null (0)	29.84 (1)	null (0)
	energy malnutrition	52.21 ± 4.72 (56)	40.18 ± 5.94 (39)	31.25 ± 1.67 (56)	35.69 ± 2.49 (39)
	protein-energy malnutrition	14.00 (1)	null (0)	42.17 (1)	null (0)
LxA	poor	50.83 ± 9.17 (6)	46.20 ± 11.32 (5)	28.70 ± 4.48 (6)	31.86 ± 3.89 (5)
	middle	48.63 ± 2.63 (161)	41.52 ± 3.81 (80)	33.03 ± 1.06 (161)	35.50 ± 1.54 (80)
	good	49.51 ± 1.59 (346)	40.56 ± 2.30 (250)	32.39 ± 0.67 (346)	35.20 ± 0.93 (250)
PMA	no risk	47.96 ± 1.92 (263)	38.03 ± 2.54 (177)	33.35 ± 0.80 (263)	35.94 ± 1.14 (177)
	low risk	49.35 ± 2.21 (171)	44.18 ± 3.37 (102)	31.93 ± 0.92 (171)	34.48 ± 1.32 (102)
	moderate risk	54.47 ± 5.42 (32)	44.31 ± 7.40 (26)	30.43 ± 2.11 (32)	34.87 ± 2.87 (26)
	high risk	52.51 ± 4.79 (47)	44.14 ± 8.38 (29)	31.75 ± 1.98 (47)	33.57 ± 2.42 (29)
PMAC	< 25th	48.51 ± 2.79 (98)	34.75 ± 3.21 (105)	33.79 ± 1.35 (98)	37.66 ± 1.39 (105)
	≥ 25th, < 50th	46.46 ± 2.55 (135)	40.51 ± 3.88 (75)	32.98 ± 1.04 (135)	34.50 ± 1.71 (75)
	≥ 50th, < 75th	49.23 ± 2.74 (143)	45.53 ± 3.80 (79)	32.59 ± 1.11 (143)	34.01 ± 1.56 (79)
	≥ 75th	52.48 ± 2.65 (135)	45.14 ± 4.58 (73)	31.26 ± 1.04 (135)	33.93 ± 1.56 (73)
IDM	< 25th	49.21 ± 2.68 (131)	42.57 ± 5.71 (46)	32.69 ± 1.12 (131)	36.12 ± 2.16 (46)
	≥ 25th, < 50th	47.86 ± 2.59 (123)	44.28 ± 5.63 (50)	33.33 ± 1.05 (123)	34.53 ± 2.34 (50)
	≥ 50th, < 75th	46.64 ± 3.55 (95)	38.39 ± 4.17 (75)	33.74 ± 1.36 (95)	35.63 ± 1.88 (75)
	≥ 75th	52.65 ± 4.01 (65)	42.28 ± 3.51 (89)	31.43 ± 1.52 (65)	34.39 ± 1.39 (89)
VBD	adequate vit. B status	48.00 ± 1.25 (666)	40.39 ± 1.60 (497)	32.89 ± 0.54 (666)	34.91 ± 0.65 (497)
	functional vit. B deficiency	50.36 ± 2.31 (182)	41.34 ± 4.04 (85)	32.29 ± 0.93 (182)	34.82 ± 1.56 (85)
GLIM	Clean undernutrition	48.38 ± 12.33 (13)	46.50 ± 26.46 (2)	35.58 ± 4.82 (13)	33.98 ± 4.96 (2)
	DRM without inflammation	51.40 ± 5.40 (35)	61.25 ± 19.26 (4)	33.82 ± 2.76 (35)	27.56 ± 9.72 (4)
	DRM with inflammation	62.50 ± 21.01 (4)	57.00 (1)	29.41 ± 4.67 (4)	31.15 (1)
	BMI high	47.57 ± 1.22 (680)	40.59 ± 1.62 (499)	33.00 ± 0.51 (680)	34.92 ± 0.66 (499)

Notes: Variables are reported as mean ± standard deviation (number of cases). **Abbreviations:** mH, metres of height; ABW, actual body weight; BMI, body mass index; ASAPS, American society of anesthesiologists' classification of physical status; ODI, Oswestry disability index; PH, physical health summary measure; CRP, C-reactive protein; AHB, actual haemoglobin; MCV, mean corpuscular volume; MCH, mean corpuscular haemoglobin; MCHC, mean corpuscular haemoglobin concentration; NEUC, neutrophils count; LYMC, lymphocytes count; PALB, prealbumin; ALB, albumin.

Table S9. The malnutritional status, disability, and functional status in the 493 older adults.

		**ODI**		**PH**	
		Female (295)	Male (198)	Female (295)	Male (198)
BWd	undernutrition	46.00 ± 12.73 (2)	37.00 ± 18.38 (2)	35.54 ± 13.35 (2)	33.52 ± 6.49 (2)
	normal	53.91 ± 12.26 (22)	43.63 ± 14.41 (27)	31.27 ± 6.11 (22)	34.13 ± 5.12 (27)
	overnutrition	53.51 ± 16.62 (115)	41.00 ± 17.39 (56)	29.82 ± 5.59 (115)	32.82 ± 7.39 (56)
GNRI	absent nutrition risk	54.12 ± 16.03 (84)	42.49 ± 15.98 (57)	29.81 ± 5.41 (84)	33.74 ± 7.00 (57)
	low nutrition risk	64.00 ± 14.81 (7)	47.00 ± 6.08 (3)	28.44 ± 7.67 (7)	30.46 ± 1.56 (3)
	moderate nutrition risk	null (0)	null (0)	null (0)	null (0)
	major nutrition risk	null (0)	null (0)	null (0)	null (0)
INA	well-nourished	53.01 ± 16.73 (138)	43.67 ± 16.07 (87)	30.25 ± 6.20 (138)	33.93 ± 6.94 (87)
	protein malnutrition	null (0)	null (0)	null (0)	null (0)
	energy malnutrition	57.00 ± 14.56 (29)	44.93 ± 14.45 (29)	28.79 ± 6.39 (29)	33.03 ± 6.28 (29)
	protein-energy malnutrition	null (0)	null (0)	null (0)	null (0)
LxA	poor	61.50 ± 12.46 (8)	45.00 ± 12.08 (7)	28.07 ± 4.72 (8)	30.07 ± 6.81 (7)
	middle	57.59 ± 15.47 (56)	44.96 ± 14.60 (49)	28.86 ± 5.58 (56)	34.20 ± 5.66 (49)
	good	50.99 ± 16.67 (103)	43.07 ± 16.92 (60)	30.76 ± 6.59 (103)	33.73 ± 7.53 (60)
PMA	no risk	51.03 ± 17.08 (76)	42.92 ± 16.48 (61)	30.86 ± 7.04 (76)	34.59 ± 6.94 (61)
	low risk	56.52 ± 16.42 (52)	45.37 ± 15.12 (30)	29.24 ± 5.38 (52)	32.21 ± 6.13 (30)
	moderate risk	54.83 ± 15.42 (18)	53.12 ± 13.04 (8)	29.52 ± 5.27 (18)	30.08 ± 6.50 (8)
	high risk	55.48 ± 14.05 (21)	41.06 ± 13.80 (17)	29.11 ± 5.86 (21)	34.87 ± 6.85 (17)
PMAC	< 25th	40.00 ± 16.12 (19)	45.08 ± 17.81 (26)	34.45 ± 8.28 (19)	34.21 ± 6.73 (26)
	≥ 25th, < 50th	53.17 ± 16.35 (47)	38.38 ± 13.91 (24)	29.62 ± 6.87 (47)	37.54 ± 6.18 (24)
	≥ 50th, < 75th	56.45 ± 14.22 (38)	45.89 ± 16.98 (27)	29.71 ± 4.31 (38)	31.53 ± 6.14 (27)
	≥ 75th	56.35 ± 16.01 (62)	45.38 ± 13.83 (39)	29.15 ± 5.61 (62)	32.51 ± 6.75 (39)
IDM	< 25th	48.83 ± 14.44 (35)	35.56 ± 14.85 (9)	31.22 ± 5.73 (35)	33.83 ± 6.31 (9)
	≥ 25th, < 50th	49.92 ± 16.78 (40)	28.20 ± 8.70 (5)	29.59 ± 5.75 (40)	37.24 ± 4.65 (5)
	≥ 50th, < 75th	56.35 ± 15.11 (37)	44.17 ± 13.99 (23)	30.25 ± 6.21 (37)	33.12 ± 7.01 (23)
	≥ 75th	60.50 ± 14.91 (26)	43.15 ± 17.67 (48)	29.36 ± 5.49 (26)	32.79 ± 6.80 (48)
VBD	adequate vit B status	52.04 ± 15.77 (199)	42.09 ± 16.08 (147)	30.21 ± 6.05 (199)	33.41 ± 7.54 (147)
	functional vit B deficiency	54.93 ± 15.70 (96)	45.51 ± 16.18 (51)	29.61 ± 6.09 (96)	31.61 ± 5.87 (51)
GLIM	clean undernutrition	37.33 ± 25.42 (3)	null (0)	37.83 ± 8.40 (3)	null (0)
	DRM with inflammation	48.40 ± 21.80 (5)	55.33 ± 11.02 (3)	34.44 ± 8.60 (5)	29.31 ± 0.36 (3)
	DRM without inflammation	53.90 ± 15.05 (29)	47.56 ± 16.02 (9)	29.98 ± 5.15 (29)	35.61 ± 7.42 (9)
	BMI high	52.66 ± 16.11 (209)	41.95 ± 16.37 (155)	29.90 ± 5.83 (209)	32.94 ± 7.18 (155)

Notes: Variables are reported as mean ± standard deviation (number of cases). **Abbreviations:** ODI, Oswestry disability index; PH, physical health summary measure; BWd, body weight difference; GNRI, geriatric nutritional risk index; INA, instant nutritional assessment; LxA, combination of lymphocyte count and albumin; PMA, protein malnutrition with acute inflammation; PMAC, protein malnutrition with acute and chronic inflammation; IDM, iron deficit malnutrition; VBM, vitamin B deficit malnutrition; GLIM, global leadership initiative on malnutrition; DRM, disease-related malnutrition; BMI, body mass index.
